# Suggestions as to a Method of Closing Recto-Vaginal Fistula

**Published:** 1876-10

**Authors:** Julius F. Miner

**Affiliations:** Professor Special and Clinical Surgery Buffalo Medical College


BUFFALO

### Medical and Surgical Journal.

VOL XVI. OCTOBER, 1876. No. 3.

### Original Communications.

———:o:———

ART. I.—*Suggestions as to a Method of Closing recto-vaginal fistula.* By JULIUS F. MINER, M. D., Professor Special and Clinical Surgery Buffalo Medical College.

Recto-vaginal Fistula, after a vast amount of trial, was abandoned by the older surgeons as incurable. The success attending the introduction of metal suture, and the successful closure of vesico-vaginal fistula by improved methods, revived the expectations that similar results would obtain in recto-vaginal as in vesico-vaginal fistula, but I think the experienced surgeons of the present day have found much greater difficulty in closing fistula of the recto-vaginal septum than in closing vesico-vaginal fistula. This, at least, has been my own experience, and I am now speaking of what I know from personal observation.

I have tried to close recto-vaginal fistula and failed. I have operated upon patients who have been previously operated upon by some of the most experienced and capable surgeons of this country, who, after repeated trials have failed; I was nothing more successful. From disappointment and failure I now began to canvass the possibilities. Could recto-vaginal fistula be closed? Do surgeons know of any method of operation offering reasonable chances of success?

I have practiced all the more approved plans of proceedure; I have seen my friends make similar attempts, and we have all alike failed. I might attribute my own failure to want of experience in the operation, but taking the observation as a whole, such conclusion is untenable. My recent observations embody the suggestion which I desire to make.

The anatomical characters of the rectum are well known; the rectum is a thick, firm, fibrous sheath, loosly attached upon its external surface to adjacent tissues, capable of distension and also of *displacement.*

It now occured to me that I could detach the rectum from its connections and draw it down, cutting off all below the fissure or opening, thus making the canal *new* rather than longer trying to mend it, and could stitch the shortened tube to the verge of the anus, or could, at least, make a flap of the rectal tissue and draw it down so as to insure the closure of the fistula. Fecal matter retained in the lower rectum, constantly finding its way through the opening appeared the principal cause of failure, a condition which rupture or distention of the anal sphincter did not prove capable of preventing. If the tube could be drawn down it would be impervious, and there conld be no such source of failure. It is, perhaps, not necessary, possibly it is better, not to separate the rectum in its whole extent, but only to cut up an adequate flap and fully cover the fistula. As is well known, these openings are generally near the outlet, situated exactly where it is most liable to have contents of the bowels forced through into the vagina. When higher up, they are a little more difficult to reach, but easier to cure.

The few opportunities I have had to test my suggestion justifies the conclusion that recto-vaginal fistula can be closed by this plan of procedure more certainly than by any other, and I am quite sure surgeons will have opportunity to try this after all former methods have failed.

